# Comparative analysis of inflammatory signature profiles in eosinophilic and noneosinophilic chronic rhinosinusitis with nasal polyposis

**DOI:** 10.1042/BSR20193101

**Published:** 2020-02-24

**Authors:** Yao Yao, Chunguang Yang, Xing Yi, Shaobing Xie, Hong Sun

**Affiliations:** 1 Department of Otolaryngology Head and Neck Surgery, Xiangya Hospital of Central South University, Changsha, Hunan, China; 2 Province Key Laboratory of Otolaryngology Critical Diseases, Changsha, Hunan, China

**Keywords:** antibody array, bioinformatics analysis, chronic rhinosinusitis with nasal polyposis, endotype, eosinophil, inflammation

## Abstract

Chronic rhinosinusitis with nasal polyposis (CRSwNP) represents a heterogeneous disorder that can be classified into either eosinophilic or noneosinophilic endotypes. However, the immunological mechanisms of each remain unclear. The purpose of the present study was to compare and analyze inflammatory signatures of eosinophilic CRSwNP (ECRSwNP) and noneosinophilic CRSwNP (NECRSwNP). Cytokine antibody array was used to identify inflammatory mediators that were differentially expressed among ECRSwNP, NECRSwNP, and control groups. Then, bioinformatics approaches were conducted to explore biological functions and signaling pathways. In addition, pairwise correlation analyses were performed among differential levels of inflammatory mediators and tissue eosinophil infiltration. The results showed that nine mediators were significantly up-regulated in ECRSwNP, including eotaxin-2, eotaxin-3, CCL18, IL-4, IL-5, IL-10, IL-12p70, IL-13, and IL-15. Bioinformatics analysis indicated that these mediators were mainly enriched in leukocyte chemotaxis and proliferation, JAK-STAT cascade, asthma, and Th1 and Th2 cell differentiation. Furthermore, seven mediators were identified to be significantly up-regulated in NECRSwNP, including CCL20, resistin, transforming growth factor (TGF)-β2, triggering receptor expressed on myeloid cells 1 (TREM-1), CD14, glucocorticoid-induced tumor necrosis factor receptor related protein (GITR), and lipocalin-2. These mediators were closely associated with LPS responses, neutrophil chemotaxis and migration, and IL-17 signaling pathway. In addition, pairwise correlation analyses indicated that differential levels of inflammatory mediators in ECRSwNP and NECRSwNP were broadly correlated with each other and with tissue eosinophil infiltration. In conclusion, we found that ECRSwNP and NECRSwNP exhibited different patterns of inflammatory signatures. These findings may provide further insights into heterogeneity of CRSwNP.

## Introduction

Chronic rhinosinusitis with nasal polyposis (CRSwNP) is a complex inflammatory condition characterized by the presence of edematous masses of inflamed mucosa in the upper airways [1,2]. CRSwNP has a considerable impact on patients’ quality of life and is associated with high cost of management and significant morbidity [3]. Eosinophilic inflammation has been recognized as a major pathologic hallmark of CRSwNP in Caucasians. In contrast, recent studies have indicated that over half of the patients with CRSwNP in East Asia exhibit noneosinophilic inflammation [4,5]. Due to this heterogeneity, CRSwNP can be divided into two distinct endotypes, namely eosinophilic CRSwNP (ECRSwNP) and noneosinophilic CRSwNP (NECRSwNP) [6,7]. Nevertheless, the precise mechanisms that underlie the pathogenesis of these two endotypes are still largely unknown.

Conceptually, inflammatory mediators are small biologically active molecules that are important in the regulation of the growth, differentiation, and activation of immune cells at inflamed sites. Therefore, the mechanisms of inflammation in ECRSwNP and NECRSwNP involve a large number of inflammatory mediators and their associated pathways. Identifying and quantifying differentially expressed inflammatory mediators between ECRSwNP and NECRSwNP may expand our understanding of CRSwNP heterogeneity and aid to develop novel biological therapies [8–10]. Unfortunately, only few studies have compared inflammatory signatures between these two endotypes [11–13]. In addition, previous studies have been restricted by the limited range of inflammatory mediators tested.

Thus, to address this issue, a more comprehensive comparative analysis is needed. Antibody array is a high-throughput technique that allows detection of numerous inflammatory mediators on one membrane simultaneously. Hence, in the present study, we used a cytokine antibody array to delineate more complete inflammatory signature profiles for ECRSwNP and NECRSwNP and then employed bioinformatics tools to identify key biological functions and signaling pathways related to differential levels of inflammatory mediators.

## Materials and methods

### Patients and biopsy specimens

Adult subjects, including 30 patients with CRSwNP and 10 controls, were recruited from the Department of Otolaryngology Head and Neck Surgery of Xiangya Hospital of Central South University, Changsha, China. The diagnosis of CRSwNP was established by medical history, nasal endoscopy, and computed tomography (CT) scan of the sinuses according to the European Position Paper on Rhinosinusitis and Nasal Polyps 2012 guidelines (EPOS 2012) [1]. Subjects with an immune deficient disease, antrochoanal polyps, fungal rhinosinusitis, cystic fibrosis, or primary ciliary dyskinesia were excluded from the study. Patients without sinus disease undergoing septoplasty or endoscopic skull base surgery were enrolled as controls. Any systemic or nasal administration of corticosteroids or antibiotics were ceased in all subjects 4 weeks preoperatively. Atopic status was assessed by using skin prick tests to a standard panel of common allergens, and patients were given a diagnosis of asthma by a pneumologist based on medical history and airway responsiveness testing. Preoperative CT scans were scored using the Lund-Mackay staging system [14]. Polyps were graded using the 0 to 3 scoring system recommended by the EPOS 2012 [1]. The study was approved by the Ethics Committee of Xiangya Hospital of Central South University, and written informed consent was obtained from all subjects before enrollment in the study.

Polyp tissues and turbinate mucosa were harvested from patients with CRSwNP and control subjects during endoscopic surgery, respectively. Tissue samples were divided into two parts: 1 part was fixed in 10% formaldehyde and subsequently embedded in paraffin wax for histological staining; the other part was snap-frozen in liquid nitrogen and stored at −80°C until use for immunoassays.

### Determination of ECRSwNP and NECRSwNP

Paraffin-embedded samples were sectioned at 4 µm thickness and were stained with hematoxylin and eosin. All stained sections were examined by two independent observers who were blind to the clinical diagnosis and characteristics of the patients. The number of the infiltrating eosinophils was counted in 10 randomly selected high-power fields (HPFs). Referring to previously published criterion [4,15], CRSwNP was defined as eosinophilic when the percentage of tissue eosinophils was more than 10% of total inflammatory cells and as noneosinophilic otherwise.

### Cytokine antibody array analysis

We employed a glass chip-based multiplex sandwich ELISA system (QAH-CAA-4000; RayBiotech, Norcross, GA, U.S.A.) to measure the concentrations of 200 different human inflammatory mediators (Supplementary Table S1). This array platform was a combination of five forty-cytokine arrays and each antibody was printed in quadruplicate. Measurements were performed according to the recommended protocols from the manufacturer. Briefly, tissue samples were weighed and homogenized and then the supernatants were harvested. The protein concentration of each extract was determined and normalized to 500 μg/ml. The arrays were blocked with sample buffer and then incubated with samples or serial diluted cytokine standards overnight at 4°C. After multiple washes, the arrays were incubated with a cocktail of biotinylated antibodies. The arrays were then washed and incubated with Cy3 equivalent dye-conjugated streptavidin. The images were captured using a microarray scanner (InnoScan 300; Innopsys, Carbonne, France). The fluorescence intensity data were analyzed with the array-specific Q-Analyzer Software (RayBiotech). The results were expressed as picogram per milliliter (pg/ml).

### Statistical analysis

Statistical analyses were performed using SPSS 22.0 (SPSS, Chicago, IL) and R 3.4.0 (R Foundation for Statistical Computing, Vienna, Austria). The demographic and clinical characteristics were studied with the Fisher’s exact test for categorical variables and Mann–Whitney *U* test for continuous variables. Differential mediator expression among three groups was first analyzed using the Kruskal–Wallis *H* test followed by the Benjamini–Hochberg false discovery rate (FDR) procedure for multiple testing correction [16]. If significance was found, the Mann–Whitney *U* test was then applied for between-group comparisons. The Spearman rank test was used to determine correlations, and pairwise correlation matrix was generated using the corrplot package in R 3.4.0. In addition, principal component analysis (PCA) was performed and visualized using ggbiplot package in R 3.4.0, and mediator concentrations were log2-transformed in the analysis. A *P* value of less than 0.05 was considered statistically significant.

### Bioinformatics analysis

To explore the biological functions and signaling pathways of differentially levels of inflammatory mediators, we performed Gene Ontology Biological Process (GO-BP) and Kyoto Encyclopedia of Genes and Genomes (KEGG) enrichment analyses using the clusterProfiler package in R 3.4.0 [17]. FDR-adjusted *P* < 0.05 was set as the screening criterion.

## Results

### Patient characteristics

**Figure 1:**
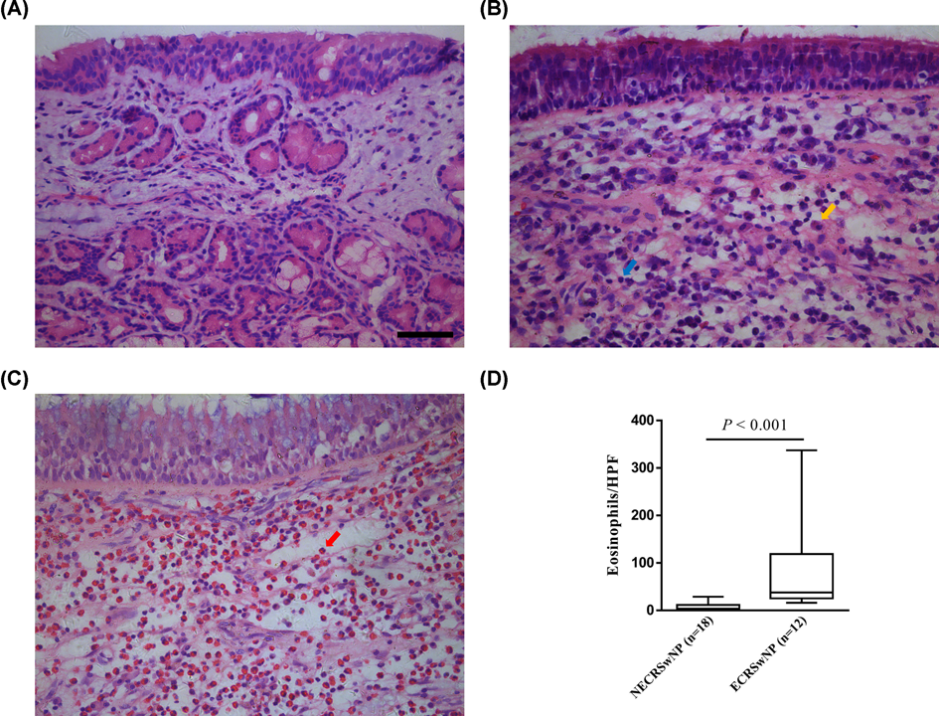
Representative hematoxylin and eosin staining of control tissues and nasal polyps (**A**) Control mucosa, (**B**) noneosinophilic polyp, and (**C**) eosinophilic polyp (Original magnification: ×400; scale bar = 50 µm; plasma cell, orange arrow; lymphocyte, blue arrow; eosinophil, red arrow). (**D**) Comparison of number of tissue eosinophils per HPF between ECRSwNP and NECRSwNP. Data are presented as a box and whisker plot, and the Mann–Whitney *U* test was used for the statistical analysis. Abbreviations: ECRSwNP, eosinophilic chronic rhinosinusitis with nasal polyposis; NECRSwNP, noneosinophilic chronic rhinosinusitis with nasal polyposis; HPF, high-power field.

The demographic and clinical characteristics of all subjects enrolled in the present study are summarized in Table 1. Among 30 CRSwNP patients, 12 (40%) were identified as ECRSwNP, and the rest (*n* = 18, 60%) were classified as NECRSwNP. The three groups were not significantly different with respect to age distribution, sex ratio, and the presence of atopy and asthma comorbidity. In addition, CT and polyp scores did not differ significantly between the ECRSwNP and NECRSwNP groups. Typical histological findings of eosinophilic and noneosinophilic polyps are shown in Figure 1. As expected, marked eosinophil infiltration was observed in ECRSwNP group (*P* < 0.001). On the other hand, most of the infiltrating cells were plasma cells and lymphocytes in the NECRSwNP group.

**Table 1:** Baseline characteristics of the subjects

	Control (*n* = 10)	NECRSwNP (*n* = 18)	ECRSwNP (*n* = 12)	P* value
Age, years, median (IQR)	44.5 (38–50.75)	39.5 (30.25–47.75)	49 (36–58.5)	0.464, 0.539, 0.172
Female/male sex	3/7	5/13	1/11	1.000, 0.293, 0.358
Atopy	0/10	6/18	4/12	0.062, 0.096, 1.000
Asthma	0/10	0/18	0/12	1.000, 1.000, 1.000
Bilateral CT score, median (IQR)	NA	16 (8–22)	19.5 (16–20.25)	NA, NA, 0.185
Bilateral polyp score, median (IQR)	NA	4 (3–5)	3.5 (2–5.25)	NA, NA, 0.573

Abbreviations: IQR, interquartile range; CT, computer tomography; ECRSwNP, eosinophilic chronic rhinosinusitis with nasal polyposis; NECRSwNP, noneosinophilic chronic rhinosinusitis with nasal polyposis; NA, not applicable.

* *P* value: NECRSwNP vs. Control, ECRSwNP vs. Control, and ECRSwNP vs. NECRSwNP, respectively. *P* values were obtained from the Fisher’s exact test (categorical variables) or Mann–Whitney *U* test (continuous variables).

### Differences in inflammatory mediator levels among the different groups

**Figure 2:**
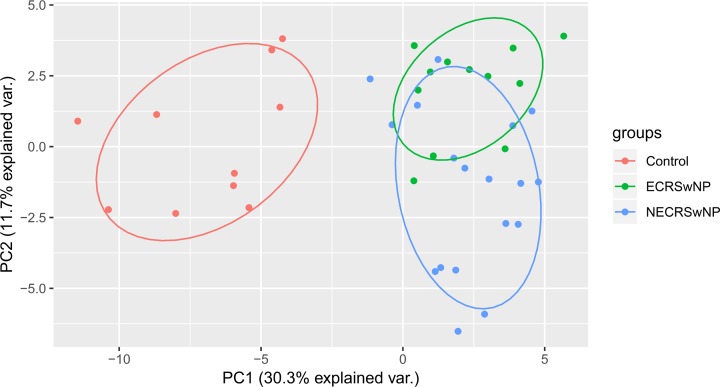
Principal component analysis based on the differential concentrations of inflammatory mediators among ECRSwNP, NECRSwNP, and control groups The *X*- and *Y*-axes represent the first and second principal components, respectively. Dots stand for individual samples. The types of the samples are color indicated. Abbreviations: ECRSwNP, eosinophilic chronic rhinosinusitis with nasal polyposis; NECRSwNP, noneosinophilic chronic rhinosinusitis with nasal polyposis.

First, the Kruskal–Wallis *H* tests were conducted to assess whether there were any overall significant differences in the levels of inflammatory mediators among ECRSwNP, NECRSwNP, and control groups. In this analysis, 44% (88/200) of the mediators were found to be statistically significant. After Benjamini–Hochberg correction for multiple comparisons, 33% (66/200) of the mediators remained significantly different (Supplementary Table S1). Of these 66 mediators, PCA was performed to gain an overview of the differences and similarities among three groups. The results demonstrated that ECRSwNP and NECRSwNP groups separated clearly from the control group on the first principal component, while there was a partial overlap between ECRSwNP and NECRSwNP samples on the second principal component (Figure 2).

Next, the Mann–Whitney *U* tests were carried out to ascertain which pairs of groups differed significantly. The results of between-group comparisons of inflammatory mediator levels are summarized in Supplementary Table S2. A total of 56 mediators were found to be significantly different in ECRSwNP when compared with controls, 57 to be significantly different in NECRSwNP versus controls, and 16 to be significantly different in ECRSwNP versus NECRSwNP.

**Figure 3:**
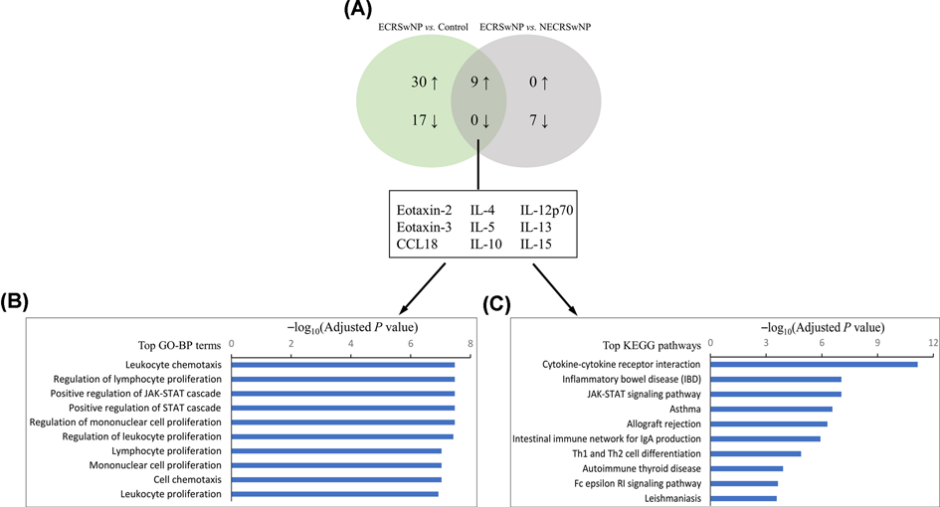
GO-BP and KEGG enrichment analyses of differential concentrations of inflammatory mediators in ECRSwNP in comparison with NECRSwNP and controls (**A**) Venn diagram summarizing the number of differential concentrations of inflammatory mediators identified in ECRSwNP when compared with NECRSwNP and controls. The up- and down-arrows represent up- and down-regulated mediators, respectively. The diagram displays the names of these differential concentrations of inflammatory mediators. (**B**) Top over-represented GO-BP terms. (**C**) Top enriched KEGG pathways. Abbreviations: ECRSwNP, eosinophilic chronic rhinosinusitis with nasal polyposis; NECRSwNP, noneosinophilic chronic rhinosinusitis with nasal polyposis; GO-BP, Gene Ontology Biological Process; KEGG, Kyoto Encyclopedia of Genes and Genomes.

**Figure 4:**
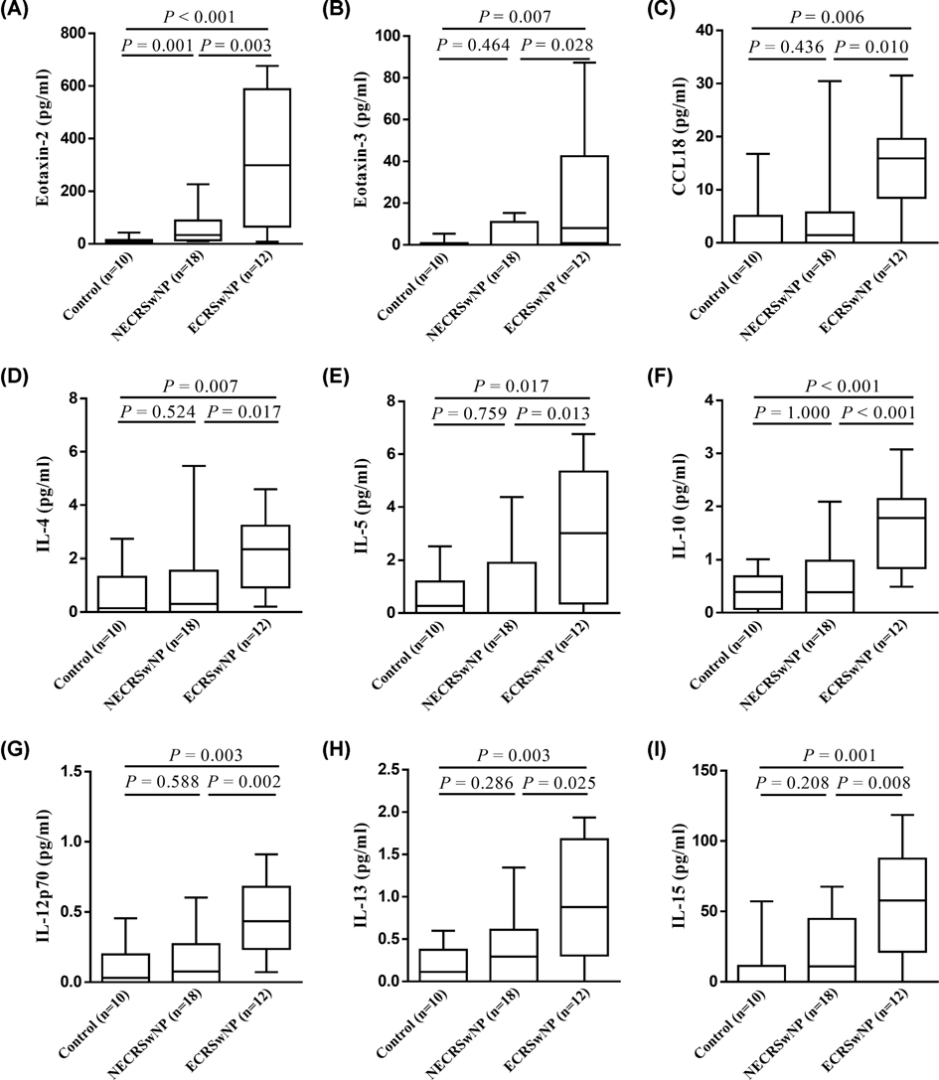
Protein levels of nine inflammatory mediators that were significantly elevated in ECRSwNP as compared with NECRSwNP and controls (**A**) Eotaxin-2, (**B**) eotaxin-3, (**C**) CCL18, (**D**) IL-4, (**E**) IL-5, (**F**) IL-10, (**G**) IL-12p70, (**H**) IL-13, and (**I**) IL-15. Data are presented as box and whisker plots, and the Mann–Whitney *U* test was used for the statistical analysis. Abbreviations: ECRSwNP, eosinophilic chronic rhinosinusitis with nasal polyposis; NECRSwNP, noneosinophilic chronic rhinosinusitis with nasal polyposis.

**Figure 5:**
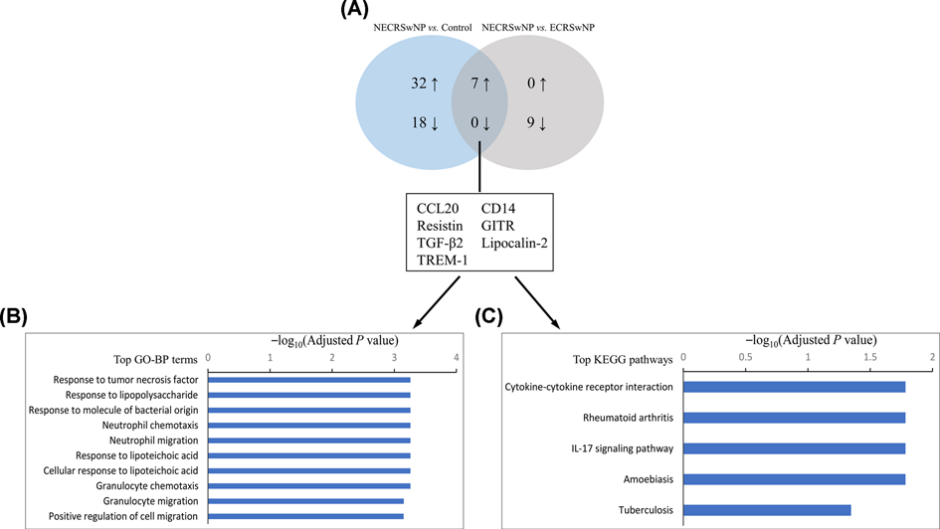
GO-BP and KEGG enrichment analyses of differential concentrations of inflammatory mediators in NECRSwNP in comparison with ECRSwNP and controls (**A**) Venn diagram summarizing the number of differential concentrations of inflammatory mediators identified in NECRSwNP when compared with ECRSwNP and controls. The up- and down-arrows represent up- and down-regulated mediators, respectively. The diagram displays the names of these differential concentrations of inflammatory mediators. (**B**) Top over-represented GO-BP terms. (**C**) Top enriched KEGG pathways. Abbreviations: ECRSwNP, eosinophilic chronic rhinosinusitis with nasal polyposis; NECRSwNP, noneosinophilic chronic rhinosinusitis with nasal polyposis; TGF-β2, transforming growth factor beta 2; TREM-1, triggering receptor expressed on myeloid cells 1; GITR, glucocorticoid-induced tumor necrosis factor receptor related protein; GO-BP, Gene Ontology Biological Process; KEGG, Kyoto Encyclopedia of Genes and Genomes.

**Figure 6:**
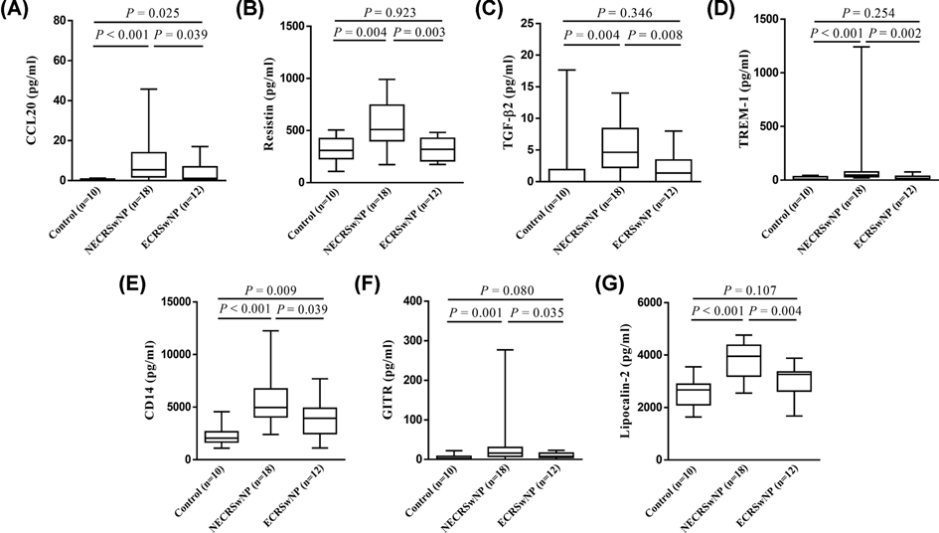
Protein levels of seven inflammatory mediators that were significantly elevated in NECRSwNP as compared with ECRSwNP and controls (**A**) CCL20, (**B**) resistin, (**C**) TGF-β2, (**D**) TREM-1, (**E**) CD14, (**F**) GITR, and (**G**) lipocalin-2. Data are presented as box and whisker plots, and the Mann–Whitney *U* test was used for the statistical analysis. Abbreviations: TGF-β2, transforming growth factor beta 2; TREM-1, triggering receptor expressed on myeloid cells 1; GITR, glucocorticoid-induced tumor necrosis factor receptor related protein; ECRSwNP, eosinophilic chronic rhinosinusitis with nasal polyposis; NECRSwNP, noneosinophilic chronic rhinosinusitis with nasal polyposis.

Finally, Venn analyses were used to determine inflammatory characteristics that were distinct in ECRSwNP and NECRSwNP. The results showed that nine mediators were significantly up-regulated in ECRSwNP as compared with NECRSwNP and controls (Figure 3A), including eotaxin-2, eotaxin-3, CCL18, IL-4, IL-5, IL-10, IL-12p70, IL-13, and IL-15 (Figure 4). Moreover, seven mediators were identified to be significantly up-regulated in NECRSwNP when compared with ECRSwNP and controls (Figure 5A), including CCL20, resistin, transforming growth factor (TGF)-β2, triggering receptor expressed on myeloid cells 1 (TREM-1), CD14, glucocorticoid-induced tumor necrosis factor receptor related protein (GITR), and lipocalin-2 (Figure 6).

### Functional and pathway enrichment analysis

To ascertain the different immunopathologic mechanisms of ECRSwNP and NECRSwNP, distinct inflammatory signature profiles in each endotype were subjected to functional enrichment and pathway analysis, respectively. In the analysis of inflammatory signature profile in ECRSwNP, the significantly over-represented GO-BP terms included leukocyte chemotaxis and proliferation and JAK-STAT cascade (Figure 3B). The significantly enriched KEGG pathways contained cytokine–cytokine receptor interaction, JAK-STAT signaling pathway, asthma, and Th1 and Th2 cell differentiation (Figure 3C). As for inflammatory signature profile in NECRSwNP, GO-BP enrichment analysis demonstrated that response to lipopolysaccharide (LPS), response to molecule of bacterial origin, and neutrophil chemotaxis and migration were significantly over-represented (Figure 5B). The significantly enriched KEGG pathways included cytokine–cytokine receptor interaction and IL-17 signaling pathway (Figure 5C).

### Correlations among inflammatory mediators and tissue eosinophil infiltration

**Figure 7:**
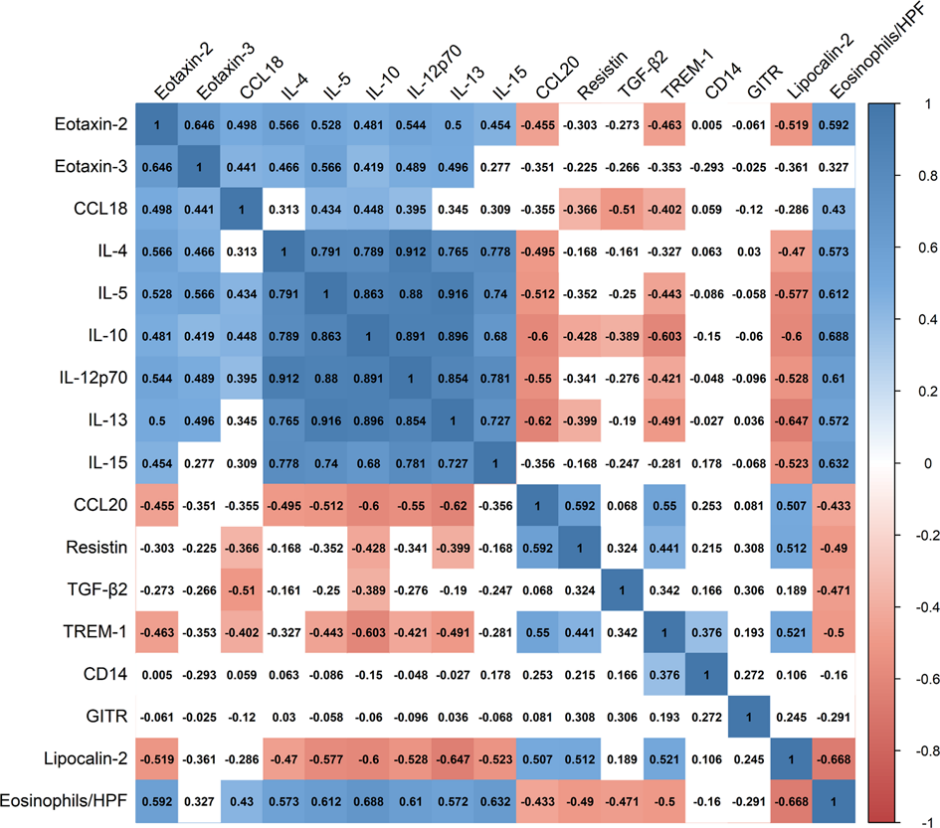
Correlation matrix showing pairwise associations of inflammatory mediators and tissue eosinophil infiltration Color bar represents *r* values. Positive and negative correlations are indicated by blue and red colors, respectively. Non-significant correlations are uncolored. Abbreviations: TGF-β2, transforming growth factor beta 2; TREM-1, triggering receptor expressed on myeloid cells 1; GITR, glucocorticoid-induced tumor necrosis factor receptor; HPF, high-power field.

We further assessed if distinct inflammatory signature profiles in ECRSwNP and NECRSwNP could correlate with one another and with tissue eosinophil infiltration. To do so, we performed pairwise correlation analyses among these variables (Figure 7). The results showed that the levels of inflammatory mediators were broadly associated with each other in all CRSwNP subjects. The highest positive correlation was between IL-5 and IL-13 (*r* = 0.916, *P* < 0.001), and the highest negative correlation was between IL-13 and lipocalin-2 (*r* = −0.647, *P* < 0.001). Furthermore, the levels of inflammatory mediators were generally correlated with tissue eosinophil infiltration. For instance, IL-15 concentrations were positively correlated with tissue eosinophil numbers (*r* = 0.632, *P* < 0.001), and TREM-1 levels were inversely associated with tissue eosinophil counts (*r* = −0.500, *P* = 0.005). Overall, these findings reflected complex interactions among differential levels of inflammatory mediators and tissue eosinophil infiltration.

## Discussion

ECRSwNP and NECRSwNP represent two distinct endotypes of CRSwNP that exhibit different clinical and pathologic features [18–21]. For better management of ECRSwNP and NECRSwNP, there is a clear need to investigate the underlying different molecular mechanisms of these two endotypes. Differences in inflammatory signature profiles between ECRSwNP and NECRSwNP may play a critical role in CRSwNP heterogeneity. Therefore, in the present study, we performed multi-analyte profiling of various immune factors, characterized distinct inflammatory characteristics for ECRSwNP and NECRSwNP, and tested for correlations with tissue eosinophil infiltration.

In the analysis of cytokine antibody array data, a total of nine inflammatory mediators were identified to be markedly up-regulated in ECRSwNP in comparison with NECRSwNP and controls. Many of them are classical Th2‐related cytokines and chemokines including IL-4, IL-5, IL-13, eotaxin-2, eotaxin-3, and CCL18, which can together promote the maturation, activation, and recruitment of eosinophils to polyp tissues [22–24]. Indeed, our pairwise correlation analyses also showed significantly positive associations of these mediator levels with each other and with mucosal eosinophilia. Thus, these results confirmed an exaggerated Th2 inflammatory signature in ECRSwNP, which are in agreement with previous literature [4,12,25].

It should be noted that the protein levels of IL-10, IL-12p70, and IL-15 were also significantly elevated in ECRSwNP. IL-10 is a potent anti-inflammatory cytokine that exerts immunosuppressive functions to limit inappropriate inflammatory responses [26]. As IL-12p70 can efficiently stimulate the differentiation of naive CD4^+^ T cells to the Th1 subset, IL-12p70 has been described as an inhibitory factor for Th2 induction and development [27,28]. IL-15 is a pleiotropic cytokine that has immunomodulatory effects on diverse cell types [29]. Recently, several *in vivo* studies have demonstrated a protective effect of IL-15 in Th2-mediated eosinophilic airway inflammation [30]. Altogether, it is tempting to speculate that IL-10, IL-12p70, and IL-15 may play negative feedback roles in resistance to an excessive Th2 inflammatory reaction in ECRSwNP. Yet, further studies are necessary to assess this hypothesis.

Although NECRSwNP is a more prevalent endotype of CRSwNP in East Asian population [4,5], few publications have focused on the immunological characteristics of noneosinophilic nasal polyps. In the present study, we found a total of seven inflammatory mediators to be significantly up-regulated in NECRSwNP compared with ECRSwNP and controls, including CCL20, resistin, TGF-β2, TREM-1, CD14, GITR, and lipocalin-2. The roles of most of these mediators have not been characterized before in NECRSwNP. Using GO-BP and KEGG enrichment analyses, we found that these inflammatory mediators were mainly concentrated in LPS responses, neutrophil chemotaxis and migration, and IL-17 signaling pathway. In addition, our pairwise correlation analyses suggested that the concentrations of these mediators were generally negatively associated with polyp eosinophil numbers.

LPS is a ubiquitous cell wall constituent of gram-negative bacteria. CD14, an important receptor in the innate immune system, plays a pivotal role in sensing and binding of LPS [31,32]. Elevated protein level of CD14 in noneosinophilic nasal polyps might be indicative of high LPS exposure in NECRSwNP [33]. Importantly, it has been documented that the expression of resistin, TREM-1, lipocalin-2, and CCL20 are strongly up-regulated upon stimulation with LPS [34–38]. Resistin has the potential to induce neutrophil proinflammatory responses and promote neutrophil extracellular trap (NET) formation [39]. TREM-1, expressed on neutrophils and monocytes/macrophages, has been shown to amplify innate immune responses via triggering neutrophil degranulation and enhancing phagocytic activity [36,40,41]. Furthermore, lipocalin-2, also known as neutrophil gelatinase-associated lipocalin (NGAL), has a bacteriostatic effect in the innate immune responses against invading pathogens [37,42]. CCL20 is expressed on epithelial cells and can function as a potent chemotactic factor for the recruitment of CCR6-expressing Th17 cells to sites of inflammation [38,43,44]. Collectively, our findings indicated that NECRSwNP is highly related to neutrophilic Th17 inflammatory responses to LPS exposure.

Some limitations in the present study should be acknowledged. First, it was designed as a cross-sectional study, in which dynamic changes of immune mediator levels in the occurrence and development of ECRSwNP and NECRSwNP could not be determined and compared. Second, the sample size of the present study was relatively small. This may limit statistical power to reveal differences in inflammatory mediator levels among ECRSwNP, NECRSwNP, and control groups. Thus, further investigation in another independent larger cohort is required to confirm the findings of the present study.

## Conclusions

In summary, using the antibody array technique, we found that ECRSwNP and NECRSwNP presented distinct types of inflammatory signature profiles. ECRSwNP is characterized by a predominant Th2 milieu, whereas NECRSwNP is closely associated with LPS responses and neutrophilic Th17-driven inflammation. The results of the present study may contribute to a better understanding of CRSwNP heterogeneity and provide new insight into the development of personalized therapeutic strategies for patients with CRSwNP.

## Supplementary Material

Supplementary Tables S1 and S2

## Abbreviations

CRSwNP: chronic rhinosinusitis with nasal polyposis
ECRSwNP: eosinophilic chronic rhinosinusitis with nasal polyposis
FDR: false discovery rate
GITR: glucocorticoid-induced tumor necrosis factor receptor related protein
GO-BP: Gene Ontology Biological Process
HPF: high-power field
KEGG: Kyoto Encyclopedia of Genes and Genomes
NECRSwNP: noneosinophilic chronic rhinosinusitis with nasal polyposis
NET: neutrophil extracellular trap
PCA: principal component analysis
TGF-β2: transforming growth factor beta 2
TREM-1: triggering receptor expressed on myeloid cells 1

## References

[1] Fokkens W.J., Lund V.J., Mullol J., Bachert C., Alobid I., Baroody F. et al. (2012) European Position Paper on Rhinosinusitis and Nasal Polyps 2012. Rhinol. Suppl. 23, 3 p preceding table of contents, 1-298 22764607

[2] Orlandi R.R., Kingdom T.T., Hwang P.H., Smith T.L., Alt J.A., Baroody F.M. et al. (2016) International Consensus Statement on Allergy and Rhinology: Rhinosinusitis. Int. Forum Allergy Rhinol. 6, S22–S209 10.1002/alr.21695_c 26889651

[3] Stevens W.W., Schleimer R.P. and Kern R.C. (2016) Chronic Rhinosinusitis with Nasal Polyps. J. Allergy Clin. Immunol. Pract. 4, 565–572 10.1016/j.jaip.2016.04.012 27393770 PMC4939220

[4] Cao P.P., Li H.B., Wang B.F., Wang S.B., You X.J., Cui Y.H. et al. (2009) Distinct immunopathologic characteristics of various types of chronic rhinosinusitis in adult Chinese. J. Allergy Clin. Immunol. 124, 478–484, 484.e471-472 10.1016/j.jaci.2009.05.017 19541359

[5] Wang X., Zhang N., Bo M., Holtappels G., Zheng M., Lou H. et al. (2016) Diversity of TH cytokine profiles in patients with chronic rhinosinusitis: A multicenter study in Europe, Asia, and Oceania. J. Allergy Clin. Immunol. 138, 1344–1353 10.1016/j.jaci.2016.05.041 27544740

[6] Akdis C.A., Bachert C., Cingi C., Dykewicz M.S., Hellings P.W., Naclerio R.M. et al. (2013) Endotypes and phenotypes of chronic rhinosinusitis: a PRACTALL document of the European Academy of Allergy and Clinical Immunology and the American Academy of Allergy, Asthma & Immunology. J. Allergy Clin. Immunol. 131, 1479–1490 23587334 10.1016/j.jaci.2013.02.036 PMC4161279

[7] Gurrola J. II and Borish L. (2017) Chronic rhinosinusitis: Endotypes, biomarkers, and treatment response. J. Allergy Clin. Immunol. 140, 1499–1508 10.1016/j.jaci.2017.10.006 29106996

[8] Kim Y.H., Nakayama T. and Bau D.T. (2017) Mediators of Allergic Asthma and Rhinosinusitis. Mediators Inflamm. 2017, 7405245 29109621 10.1155/2017/7405245 PMC5646337

[9] De Greve G., Hellings P.W., Fokkens W.J., Pugin B., Steelant B. and Seys S.F. (2017) Endotype-driven treatment in chronic upper airway diseases. Clin. Transl. Allergy 7, 22 10.1186/s13601-017-0157-8 28706720 PMC5506670

[10] Casale T.B. (2017) Biologics and biomarkers for asthma, urticaria, and nasal polyposis. J. Allergy Clin. Immunol. 139, 1411–1421 10.1016/j.jaci.2017.03.006 28477720

[11] Kim D.K. and Eun K.M. (2019) Comparison Between Signature Cytokines of Nasal Tissues in Subtypes of Chronic Rhinosinusitis. Allergy Asthma Immunol. Res. 11, 201–211 10.4168/aair.2019.11.2.201 30661312 PMC6340796

[12] Sun C., Ouyang H. and Luo R. (2017) Distinct characteristics of nasal polyps with and without eosinophilia. Braz. J. Otorhinolaryngol. 83, 66–72 10.1016/j.bjorl.2016.01.012 27166273 PMC9444718

[13] Cao P.P., Zhang Y.N., Liao B., Ma J., Wang B.F., Wang H. et al. (2014) Increased local IgE production induced by common aeroallergens and phenotypic alteration of mast cells in Chinese eosinophilic, but not non-eosinophilic, chronic rhinosinusitis with nasal polyps. Clin. Exp. Allergy 44, 690–700 10.1111/cea.12304 24597471 PMC4013492

[14] Lund V.J. and Mackay I.S. (1993) Staging in rhinosinusitus. Rhinology 31, 183–184 8140385

[15] Kim D.K., Park M.H., Chang D.Y., Eun K.M., Shin H.W., Mo J.H. et al. (2014) MBP-positive and CD11c-positive cells are associated with different phenotypes of Korean patients with non-asthmatic chronic rhinosinusitis. PLoS One 9, e111352 10.1371/journal.pone.0111352 25361058 PMC4216068

[16] Benjamini Y. and Hochberg Y. (1995) Controlling the False Discovery Rate: A Practical and Powerful Approach to Multiple Testing. J. Roy. Statist. Soc. Ser. A 57, 289–300

[17] Yu G., Wang L.G., Han Y. and He Q.Y. (2012) clusterProfiler: an R package for comparing biological themes among gene clusters. OMICS 16, 284–287 10.1089/omi.2011.0118 22455463 PMC3339379

[18] Ishitoya J., Sakuma Y. and Tsukuda M. (2010) Eosinophilic chronic rhinosinusitis in Japan. Allergol Int. 59, 239–245 10.2332/allergolint.10-RAI-0231 20657162

[19] Takeno S., Hirakawa K. and Ishino T. (2010) Pathological mechanisms and clinical features of eosinophilic chronic rhinosinusitis in the Japanese population. Allergol Int. 59, 247–256 10.2332/allergolint.10-RAI-0202 20567131

[20] Shi L.L., Xiong P., Zhang L., Cao P.P., Liao B., Lu X. et al. (2013) Features of airway remodeling in different types of Chinese chronic rhinosinusitis are associated with inflammation patterns. Allergy 68, 101–109 10.1111/all.12064 23157215

[21] Xie L., Liu A.G., Cui Y.H., Zhang Y.P., Liao B., Li N.N. et al. (2015) Expression profiles of prostaglandin E2 receptor subtypes in aspirin tolerant adult Chinese with chronic rhinosinusitis. Am. J. Rhinol. Allergy 29, 322–328 10.2500/ajra.2015.29.4205 26358341

[22] Yao Y., Xie S., Yang C., Zhang J., Wu X. and Sun H. (2017) Biomarkers in the evaluation and management of chronic rhinosinusitis with nasal polyposis. Eur. Arch. Otorhinolaryngol. 274, 3559–3566 10.1007/s00405-017-4547-2 28365802

[23] Peterson S., Poposki J.A., Nagarkar D.R., Chustz R.T., Peters A.T., Suh L.A. et al. (2012) Increased expression of CC chemokine ligand 18 in patients with chronic rhinosinusitis with nasal polyps. J. Allergy Clin. Immunol. 129, 119-127.e111-119 10.1016/j.jaci.2011.08.021 PMC3246095 21943944

[24] Li C.W., Zhang K.K., Li T.Y., Lin Z.B., Li Y.Y., Curotto de Lafaille M.A. et al. (2012) Expression profiles of regulatory and helper T-cell-associated genes in nasal polyposis. Allergy 67, 732–740 10.1111/j.1398-9995.2012.02811.x 22462754

[25] Wang W., Gao Z., Wang H., Li T., He W., Lv W. et al. (2016) Transcriptome Analysis Reveals Distinct Gene Expression Profiles in Eosinophilic and Noneosinophilic Chronic Rhinosinusitis with Nasal Polyps. Sci. Rep. 6, 26604 10.1038/srep26604 27216292 PMC4877582

[26] Ouyang W. and O’Garra A. (2019) IL-10 Family Cytokines IL-10 and IL-22: from Basic Science to Clinical Translation. Immunity 50, 871–891 10.1016/j.immuni.2019.03.020 30995504

[27] Hino A., Kweon M.N., Fujihashi K., McGhee J.R. and Kiyono H. (2004) Pathological role of large intestinal IL-12p40 for the induction of Th2-type allergic diarrhea. Am. J. Pathol. 164, 1327–1335 10.1016/S0002-9440(10)63219-1 15039220 PMC1615363

[28] Meyts I., Hellings P.W., Hens G., Vanaudenaerde B.M., Verbinnen B., Heremans H. et al. (2006) IL-12 contributes to allergen-induced airway inflammation in experimental asthma. J. Immunol. 177, 6460–6470 10.4049/jimmunol.177.9.6460 17056578

[29] Jabri B. and Abadie V. (2015) IL-15 functions as a danger signal to regulate tissue-resident T cells and tissue destruction. Nat. Rev. Immunol. 15, 771–783 10.1038/nri3919 26567920 PMC5079184

[30] Venkateshaiah S.U., Zhu X., Rajavelu P., Niranjan R., Manohar M., Verma A.K. et al. (2018) Regulatory effects of IL-15 on allergen-induced airway obstruction. J. Allergy Clin. Immunol. 141, 906.e906–917.e906 28606589 10.1016/j.jaci.2017.05.025 PMC5723242

[31] Anas A., van der Poll T. and de Vos A.F. (2010) Role of CD14 in lung inflammation and infection. Critical Care 14, 209 10.1186/cc8850 20236452 PMC2887102

[32] Zanoni I. and Granucci F. (2013) Role of CD14 in host protection against infections and in metabolism regulation. Front Cell Infect. Microbiol. 3, 32 23898465 10.3389/fcimb.2013.00032 PMC3721004

[33] Lauener R.P., Birchler T., Adamski J., Braun-Fahrländer C., Bufe A., Herz U. et al. (2002) Expression of CD14 and Toll-like receptor 2 in farmers’ and nonfarmers’ children. Lancet North Am. Ed. 360, 465–466 10.1016/S0140-6736(02)09641-1 12241724

[34] Lu S.-C., Shieh W.-Y., Chen C.-Y., Hsu S.-C. and Chen H.-L. (2002) Lipopolysaccharide increases resistin gene expression in vivo and in vitro. FEBS Lett. 530, 158–162 10.1016/S0014-5793(02)03450-6 12387885

[35] Lehrke M., Reilly M.P., Millington S.C., Iqbal N., Rader D.J. and Lazar M.A. (2004) An Inflammatory Cascade Leading to Hyperresistinemia in Humans. PLoS Med. 1, e45 10.1371/journal.pmed.0010045 15578112 PMC529430

[36] Carla Bosco M., Raggi F. and Varesio L. (2016) Therapeutic Potential of Targeting TREM-1 in Inflammatory Diseases and Cancer. Curr. Pharm. Des. 22, 6209–6233 10.2174/1381612822666160826110539 27568730

[37] Abella V., Scotece M., Conde J., Gomez R., Lois A., Pino J. et al. (2015) The potential of lipocalin-2/NGAL as biomarker for inflammatory and metabolic diseases. Biomarkers 20, 565–571 10.3109/1354750X.2015.1123354 26671823 PMC4819811

[38] Lee A.Y.S. and Körner H. (2019) The CCR6-CCL20 axis in humoral immunity and T-B cell immunobiology. Immunobiology 224, 449–454 10.1016/j.imbio.2019.01.005 30772094

[39] Jiang S., Park D.W., Tadie J.-M., Gregoire M., Deshane J., Pittet J.F. et al. (2014) Human Resistin Promotes Neutrophil Proinflammatory Activation and Neutrophil Extracellular Trap Formation and Increases Severity of Acute Lung Injury. J. Immunol. 192, 4795–4803 10.4049/jimmunol.1302764 24719460 PMC4018664

[40] Pelham C.J. and Agrawal D.K. (2014) Emerging roles for triggering receptor expressed on myeloid cells receptor family signaling in inflammatory diseases. Expert Rev. Clin. Immunol. 10, 243–256 10.1586/1744666X.2014.866519 24325404

[41] Arts R.J.W., Joosten L.A.B., van der Meer J.W.M. and Netea M.G. (2013) TREM-1: intracellular signaling pathways and interaction with pattern recognition receptors. J. Leukoc. Biol. 93, 209–215 10.1189/jlb.0312145 23108097

[42] Xiao X., Yeoh B.S. and Vijay-Kumar M. (2017) Lipocalin 2: An Emerging Player in Iron Homeostasis and Inflammation. Annu. Rev. Nutr. 37, 103–130 10.1146/annurev-nutr-071816-064559 28628361

[43] Kryczek I., Bruce A.T., Gudjonsson J.E., Johnston A., Aphale A., Vatan L. et al. (2008) Induction of IL-17+ T cell trafficking and development by IFN-gamma: mechanism and pathological relevance in psoriasis. J. Immunol. 181, 4733–4741 10.4049/jimmunol.181.7.4733 18802076 PMC2677162

[44] Lee J.H., Kang H.J., Woo J.-S., Chae S.W., Lee S.H., Hwang S.J. et al. (2006) Up-regulation of Chemokine Ligand 20 in Chronic Rhinosinusitis. JAMA Otolaryngol.–Head Neck Surgery 132, 537–541 10.1001/archotol.132.5.537 16702571

