# Supplementary material for: Comparative analysis of inflammatory signature profiles in eosinophilic and noneosinophilic chronic rhinosinusitis with nasal polyposis
Source: Biosci Rep. 2020 Feb 24;40(2):BSR20193101. doi: 10.1042/BSR20193101 (PMC7040463; doi:10.1042/BSR20193101)
Supplement: Supplementary Tables S1 and S2 [file BSR-2019-3101_supp.pdf]

Supplementary Table S1 Comparisons of 200 inflammatory mediator levels among ECRSwNP, NECRSwNP, and control groups using cytokine antibody array

Mediators	Description	Overall *P* value	Adjusted *P* value
CCL21	C-C motif chemokine 21	0.276	0.383
Axl	AXL oncogene	**0.025**	0.066
Betacellulin	Betacellulin	0.445	0.520
CCL28 (MEC)	C-C motif chemokine 28 (Mucosae-associated epithelial chemokine)	0.275	0.383
CCL27 (CTACK)	C-C motif chemokine 27 (Cutaneous T-cell-attracting chemokine)	0.586	0.634
CXCL16	C-X-C motif chemokine 16	**0.001**	**0.007**
CXCL5 (ENA-78)	C-X-C motif chemokine 5 (Epithelial-derived neutrophil-activating protein 78)	0.365	0.468
CCL26 (Eotaxin-3)	C-C motif chemokine 26	**0.007**	**0.027**
CXCL6 (GCP-2)	C-X-C motif chemokine 6 (Granulocyte chemotactic protein 2)	0.180	0.286
CXCL1 (GRO)	C-X-C motif chemokine 1 (Growth-regulated alpha protein)	**0.001**	**0.007**
CCL14 (HCC-1)	C-C motif chemokine 14	**<0.001**	**<0.001**
CCL16 (HCC-4)	C-C motif chemokine 16	**0.027**	0.070
IL-9	Interleukin 9	0.341	0.452
IL-17F	Interleukin 17F	0.097	0.188
IL-18BPα	Interleukin-18-binding protein alpha	0.900	0.909
IL-28A (IFNL2)	Interleukin 28A (Interferon lambda 2)	0.390	0.481
IL-29 (IFNL1)	Interleukin 29 (Interferon lambda 1)	0.303	0.415
IL-31	Interleukin 31	0.208	0.313
CXCL10 (IP-10)	C-X-C motif chemokine 10 (10 kDa interferon gamma-induced protein)	0.127	0.217
CXCL11 (I-TAC)	C-X-C motif chemokine 11 (Interferon-inducible T-cell alpha chemoattractant)	0.423	0.507
LIF	Leukemia inhibitory factor	0.151	0.248
TNFSF14 (LIGHT)	Tumor necrosis factor ligand superfamily member 14	0.545	0.601
XCL1 (Lymphotactin)	C motif chemokine 1	**0.017**	0.051
CCL8 (MCP-2)	C-C motif chemokine 8 (Monocyte chemotactic protein 2)	**0.003**	**0.014**

CCL7 (MCP-3)	C-C motif chemokine 7 (Monocyte chemotactic protein 3)	0.431	0.513
CCL13 (MCP-4)	C-C motif chemokine 13 (Monocyte chemotactic protein 4)	**0.030**	0.075
CCL22 (MDC)	C-C motif chemokine 22 (Macrophage-derived chemokine)	0.095	0.186
MIF	Macrophage migration inhibitory factor	0.523	0.582
CCL20 (MIP-3α)	C-C motif chemokine 20 (Macrophage inflammatory protein 3 alpha)	**<0.001**	**<0.001**
CCL19 (MIP-3β)	C-C motif chemokine 19 (Macrophage inflammatory protein 3 beta)	0.108	0.202
CCL23 (MPIF-1)	C-C motif chemokine 23 (Myeloid progenitor inhibitory factor 1)	0.275	0.383
MSP	Macrophage stimulating protein	**0.001**	**0.007**
CXCL7 (NAP-2)	C-X-C motif chemokine 7 (Neutrophil-activating peptide 2)	0.143	0.238
Osteopontin	Osteopontin	0.498	0.567
CCL18 (PARC)	C-C motif chemokine 18 (Pulmonary and activation-regulated chemokine)	**0.006**	**0.025**
Platelet factor 4	Platelet factor 4	**0.001**	**0.007**
SDF-1α	Stromal cell-derived factor 1 alpha	0.102	0.194
CCL17 (TARC)	C-C motif chemokine 17 (Thymus and activation-regulated chemokine)	0.127	0.217
CCL25 (TECK)	C-C motif chemokine 25 (Thymus-expressed chemokine)	0.421	0.507
TSLP	Thymic stromal lymphopoietin	0.576	0.626
Activin A	Activin A	**0.003**	**0.014**
AgRP	Agouti-related protein	**0.044**	0.101
Angiogenin	Angiogenin	0.221	0.323
Angiopoietin-1	Angiopoietin 1	**<0.001**	**<0.001**
Angiostatin	Angiostatin	0.142	0.238
Cathepsin S	Cathepsin S	0.676	0.708
CD40 (TNFRSF5)	CD40 (Tumor necrosis factor receptor superfamily member 5)	0.054	0.120
Cripto-1 (TDGF1)	Teratocarcinoma-derived growth factor 1	**0.042**	0.098
DAN (NBL1)	Neuroblastoma suppressor of tumorigenicity 1	0.196	0.299
DKK-1	Dickkopf-related protein 1	**0.019**	0.055

E-Cadherin	Epithelial cadherin	0.110	0.202
TROP1 (EpCAM)	Epithelial cell adhesion molecule	0.082	0.164
Fas Ligand (TNFSF6)	Fas ligand (Tumor necrosis factor ligand superfamily member 6)	0.183	0.286
FcγRIIB/C	Low affinity immunoglobulin gamma Fc region receptor II b/c	**0.007**	**0.027**
Follistatin	Follistatin	0.182	0.286
Galectin-7	Galectin-7	0.281	0.388
ICAM-2 (CD102)	Intercellular adhesion molecule 2	0.059	0.130
IL-13Rα1	Interleukin-13 receptor subunit alpha 1	0.752	0.779
IL-13Rα2	Interleukin-13 receptor subunit alpha 2	0.072	0.153
IL-17B	Interleukin 17B	**0.022**	0.060
IL-2Rα	Interleukin 2 receptor subunit alpha	**<0.001**	**<0.001**
IL-2Rβ	Interleukin 2 receptor subunit beta	**0.011**	**0.036**
IL-23	Interleukin 23	**0.004**	**0.018**
LAP (TGF-β1)	Latency-associated peptide (Transforming growth factor beta 1)	0.495	0.567
NrCAM	Neuronal cell adhesion molecule	0.311	0.420
PAI-1	Plasminogen activator inhibitor 1	**0.022**	0.060
PDGF-AB	Platelet-derived growth factor AB	**0.003**	**0.014**
Resistin	Resistin	**0.002**	**0.011**
SDF-1β	Stromal cell-derived factor 1 beta	**<0.001**	**<0.001**
gp130	Membrane glycoprotein 130	**0.037**	0.089
Shh-N	Sonic hedgehog protein N-product	**0.018**	0.053
Siglec-5 (CD170)	Sialic acid-binding Ig-like lectin 5	**0.035**	0.085
ST2 (IL-1 R4)	Interleukin 1 receptor 4	**0.030**	0.075
TGF-β2	Transforming growth factor beta 2	**0.003**	**0.014**
Tie-2	Tyrosine-protein kinase receptor TIE-2	**0.021**	0.060
Thrombopoietin	Thrombopoietin	**<0.001**	**<0.001**

TRAILR4	TNF-related apoptosis-inducing ligand receptor 4	**0.011**	**0.036**
TREM-1	Triggering receptor expressed on myeloid cells 1	**0.001**	**0.007**
VEGF-C	Vascular endothelial growth factor C	0.200	0.303
VEGFR1	Vascular endothelial growth factor receptor 1	**0.048**	0.109
Amphiregulin	Amphiregulin	0.080	0.163
BDNF	Brain-derived neurotrophic factor	0.503	0.568
bFGF	Basic fibroblast growth factor	**<0.001**	**<0.001**
BMP-4	Bone morphogenetic protein 4	0.101	0.194
BMP-5	Bone morphogenetic protein 5	0.266	0.377
BMP-7	Bone morphogenetic protein 7	0.387	0.481
β-NGF	Beta nerve growth factor	**0.039**	0.093
EGF	Epidermal growth factor	**0.001**	**0.007**
EGFR	Epidermal growth factor receptor	**0.002**	**0.011**
EG-VEGF (PK1)	Endocrine-gland-derived vascular endothelial growth factor (Prokineticin 1)	0.653	0.696
FGF-4	Fibroblast growth factor 4	0.166	0.268
FGF-7 (KGF)	Fibroblast growth factor 7 (Keratinocyte growth factor)	0.374	0.473
GDF-15	Growth differentiation factor 15	**0.001**	**0.007**
GDNF	Glial cell line-derived neurotrophic factor	0.602	0.647
Growth hormone	Growth hormone	0.251	0.361
HB-EGF	Heparin-binding EGF-like growth factor	0.547	0.601
HGF	Hepatocyte growth factor	**<0.001**	**<0.001**
IGFBP-1	Insulin-like growth factor-binding protein 1	0.122	0.216
IGFBP-2	Insulin-like growth factor-binding protein 2	**0.008**	**0.030**
IGFBP-3	Insulin-like growth factor-binding protein 3	**0.002**	**0.011**
IGFBP-4	Insulin-like growth factor-binding protein 4	0.968	0.968
IGFBP-6	Insulin-like growth factor-binding protein 6	0.370	0.471

IGF-1	Insulin-like growth factor 1	0.699	0.728
Insulin	Insulin	**0.010**	**0.035**
M-CSFR	Macrophage colony stimulating factor 1 receptor	**0.001**	**0.007**
NGFR (TNFRSF16)	Nerve growth factor receptor (Tumor necrosis factor receptor superfamily member 16)	0.388	0.481
NT-3	Neurotrophin 3	0.165	0.268
NT-4	Neurotrophin 4	0.794	0.819
Osteoprotegerin	Osteoprotegerin	0.890	0.904
PDGF-AA	Platelet-derived growth factor AA	**<0.001**	**<0.001**
PLGF	Placenta growth factor	0.663	0.702
SCF	Stem cell factor	0.568	0.621
CD117 (SCFR)	CD117 (Stem cell factor receptor)	0.385	0.481
TGF-α	Transforming growth factor alpha	0.838	0.855
TGF-β1	Transforming growth factor beta 1	0.125	0.217
TGF-β3	Transforming growth factor beta 3	0.524	0.582
VEGF-A	Vascular endothelial growth factor A	**0.006**	**0.025**
VEGFR2	Vascular endothelial growth factor receptor 2	0.404	0.493
VEGFR3	Vascular endothelial growth factor receptor 3	0.360	0.465
VEGF-D	Vascular endothelial growth factor D	0.654	0.696
CXCL13 (BLC)	C-X-C motif chemokine 13 (B lymphocyte chemoattractant)	**0.005**	**0.022**
CCL11 (Eotaxin-1)	C-C motif chemokine 11	**0.003**	**0.014**
CCL24 (Eotaxin-2)	C-C motif chemokine 24	**<0.001**	**<0.001**
G-CSF	Granulocyte colony-stimulating factor	**<0.001**	**<0.001**
GM-CSF	Granulocyte-macrophage colony-stimulating factor	**0.035**	0.085
I-309 (TCA-3/CCL1)	C-C motif chemokine 1	0.408	0.495
CD54 (ICAM-1)	CD54 (Intercellular adhesion molecule 1)	0.253	0.361
IFN-γ	Interferon gamma	0.111	0.202

IL-1α	Interleukin 1 alpha	0.218	0.323
IL-1β	Interleukin 1 beta	0.079	0.163
IL-1ra	Interleukin 1 receptor antagonist	**0.013**	**0.041**
IL-2	Interleukin 2	**0.022**	0.060
IL-4	Interleukin 4	**0.014**	**0.043**
IL-5	Interleukin 5	**0.015**	**0.045**
IL-6	Interleukin 6	0.060	0.130
IL-6R	Interleukin 6 receptor subunit alpha	0.356	0.464
IL-7	Interleukin 7	**0.023**	0.061
IL-8 (CXCL8)	Interleukin 8 (C-X-C motif chemokine 8)	0.170	0.272
IL-10	Interleukin 10	**0.001**	**0.007**
IL-11	Interleukin 11	0.052	0.117
IL-12p40	Interleukin 12 p40	0.443	0.520
IL-12p70	Interleukin 12 p70	**0.003**	**0.014**
IL-13	Interleukin 13	**0.010**	**0.035**
IL-15	Interleukin 15	**0.002**	**0.011**
IL-16	Interleukin 16	0.669	0.704
IL-17A	Interleukin 17A	**0.002**	**0.011**
CCL2 (MCP-1)	C-C motif chemokine 2 (Monocyte chemotactic protein 1)	**<0.001**	**<0.001**
M-CSF	Macrophage colony-stimulating factor 1	0.082	0.164
CXCL9 (MIG)	C-X-C motif chemokine 9 (Monokine induced by interferon gamma)	0.357	0.464
CCL3 (MIP-1α)	C-C motif chemokine 3 (Macrophage inflammatory protein 1 alpha)	0.190	0.294
CCL4 (MIP-1β)	C-C motif chemokine 4 (Macrophage inflammatory protein 1 beta)	0.126	0.217
CCL15 (MIP-1δ)	C-C motif chemokine 15 (Macrophage inflammatory protein 1 delta)	**0.010**	**0.035**
PDGF-BB	Platelet-derived growth factor BB	**0.011**	**0.036**
CCL5 (RANTES)	C-C motif chemokine 5 (Regulated upon activation, normal T cell expressed and secreted factor)	0.930	0.935

TIMP-1	Tissue inhibitor of metalloproteinases 1	0.333	0.444
TIMP-2	Tissue inhibitor of metalloproteinases 2	0.220	0.323
TNF-α	Tumor necrosis factor alpha	0.244	0.354
TNF-β	Tumor necrosis factor beta	0.499	0.567
TNFRI	Tumor necrosis factor receptor 1	**0.003**	**0.014**
TNFRII	Tumor necrosis factor receptor 2	**0.011**	**0.036**
CD137	CD137	0.087	0.172
CD166 (ALCAM)	CD166 (Activated leukocyte cell adhesion molecule)	0.074	0.156
CD80	CD80	0.803	0.824
BCMA (TNFRSF17)	B-cell maturation protein (Tumor necrosis factor receptor superfamily member 17)	0.350	0.461
CD14	CD14	**<0.001**	**<0.001**
CD30 (TNFRSF8)	CD30 (Tumor necrosis factor receptor superfamily member 8)	0.308	0.419
CD40 Ligand	CD40 ligand	**0.029**	0.074
CEACAM-1	Carcinoembryonic antigen-related cell adhesion molecule 1	**0.002**	**0.011**
DR6 (TNFRSF21)	Death receptor 6 (Tumor necrosis factor receptor superfamily member 21)	**0.023**	0.061
Dtk (TYRO3)	Tyrosine-protein kinase receptor TYRO3	0.215	0.321
CD105 (Endoglin)	CD105 (Endoglin)	**0.007**	**0.027**
ErbB3	Receptor tyrosine-protein kinase erbB-3	**<0.001**	**<0.001**
E-Selectin	E-Selectin	0.447	0.520
Fas	Apoptosis-mediating surface antigen FAS	0.110	0.202
Flt-3 Ligand	Fms-related tyrosine kinase 3 ligand	**0.008**	**0.030**
GITR (TNFRSF18)	Glucocorticoid-induced tumor necrosis factor receptor related protein (Tumor necrosis factor receptor superfamily member 18)	**0.003**	**0.014**
HVEM (TNFRSF14)	Herpes virus entry mediator A (Tumor necrosis factor receptor superfamily member 14)	0.440	0.520
CD50 (ICAM-3)	CD50 (Intercellular adhesion molecule 3)	0.148	0.245
Contactin-2	Contactin-2	0.468	0.541
IL-1R1	Interleukin 1 receptor type 1	0.109	0.202

IL-2Rγ	Interleukin 2 receptor gamma	0.191	0.294
IL-10Rβ	Interleukin 10 receptor beta	0.128	0.217
IL-17RA	Interleukin 17 receptor A	**0.014**	**0.043**
IL-21R	Interleukin 21 receptor	0.080	0.163
LIMPII	Lysosomal integral membrane protein 2	**0.002**	**0.011**
Lipocalin-2 (NGAL)	Lipocalin 2 (Neutrophil gelatinase-associated lipocalin)	**<0.001**	**<0.001**
L-Selectin	L-Selectin	**0.002**	**0.011**
LYVE-1	Lymphatic vessel endothelial hyaluronic acid receptor 1	**0.013**	**0.041**
MICA	MHC class I polypeptide-related sequence A	**0.008**	**0.030**
MICB	MHC class I polypeptide-related sequence B	0.112	0.202
NRG1-β1	Neuregulin 1 beta 1	0.117	0.209
PDGFRβ	Platelet-derived growth factor receptor beta	0.065	0.140
CD31 (PECAM-1)	CD31 (Platelet endothelial cell adhesion molecule 1)	0.515	0.579
RAGE	Receptor for advanced glycation end products	**<0.001**	**<0.001**
TIM-1 (KIM-1)	T cell immunoglobulin mucin receptor 1 (Kidney injury molecule 1)	**0.004**	**0.018**
TRAILR3	TNF-related apoptosis-inducing ligand receptor 3	**<0.001**	**<0.001**
Trappin-2	Trappin-2	0.397	0.487
uPAR	Urokinase plasminogen activator surface receptor	**<0.001**	**<0.001**
VCAM-1	Vascular cell adhesion molecule 1	**0.041**	0.096
XEDAR	X-linked ectodysplasin-A2 receptor	0.325	0.436

Overall *P* values were determined among ECRSwNP, NECRSwNP, and control groups using the Kruskal-Wallis *H* tests. Adjusted *P* values were calculated by Benjamini-Hochberg false discovery rate correction for multiple comparisons.

The significant values are indicated in bold.

Supplementary Table S2 Protein levels of 66 inflammatory mediators among ECRSwNP, NECRSwNP, and control groups

Mediators (pg/ml)	Control	NECRSwNP	ECRSwNP	*P** value
CXCL16	143.47 (122.89-158.78)	252.31 (197.12-329.94)	190.85 (171.52-261.17)	**<0.001**, **0.003**, 0.134
CCL26 (Eotaxin-3)	0 (0-0)	0 (0-8.81)	8.08 (2.67-34.2)	0.464, **0.007**, **0.028**
CXCL1 (GRO)	26.26 (15.8-63.47)	100.04 (84.91-127.67)	124.6 (51.19-188.85)	**<0.001**, **0.004**, 0.787
CCL14 (HCC-1)	237.82 (222.88-245.3)	141.68 (126.79-168.75)	158.01 (133.81-184.09)	**<0.001**, **<0.001**, 0.602
CCL8 (MCP-2)	12.16 (9.11-20.61)	0 (0-4.74)	5.26 (0-9.43)	**0.002**, **0.009**, 0.285
CCL20 (MIP-3α)	0.31 (0.04-0.74)	5.45 (2-12.17)	1.2 (0.8-3.64)	**<0.001**, **0.025**, **0.039**
MSP	0 (0-0)	13.55 (0.53-60.85)	14.45 (10.75-46.83)	**0.003**, **<0.001**, 0.662
CCL18 (PARC)	0 (0-1.86)	1.46 (0-5.52)	15.9 (11.99-19.53)	0.436, **0.006**, **0.010**
Platelet factor 4	9846.86 (7440.35-11411.69)	1172.11 (298.36-3618.96)	2953.91 (1059.65-7153.99)	**<0.001**, **0.002**, 0.215
Activin A	6.39 (0-24.18)	211.03 (141.65-318.62)	97.81 (0-278.76)	**<0.001**, 0.123, 0.172
Angiopoietin-1	366.09 (326.93-455.6)	145.55 (75.77-191.61)	160.78 (109.36-193.12)	**<0.001**, **<0.001**, 0.662
FcγRIIB/C	502.46 (407.69-812.31)	1136.36 (911.07-1400.57)	970.12 (597.32-1281.46)	**0.001**, 0.050, 0.232
IL-2Rα	9.52 (7.84-11.92)	35.72 (26.64-65.06)	55.8 (31.75-78.81)	**<0.001**, **<0.001**, 0.267
IL-2Rβ	6.89 (0.34-20.93)	54.63 (36.57-87.13)	46.01 (22.62-79.98)	**0.003**, **0.030**, 0.391
IL-23	10.86 (0.27-13.48)	28.51 (21.44-34.2)	26.07 (21.85-43.12)	**0.001**, **0.004**, 0.983
PDGF-AB	36.11 (15.18-71.58)	2.44 (0-25.07)	0.22 (0-1.01)	**0.040**, **<0.001**, 0.104
Resistin	309.96 (243.06-421.5)	509.28 (409.08-729.51)	319.09 (222.24-414.29)	**0.004**, 0.923, **0.003**
SDF-1β	2.49 (0.59-4.1)	13.83 (10.57-19.48)	8.87 (7.27-13.86)	**<0.001**, **<0.001**, 0.124
TGF-β2	0 (0-1.38)	4.63 (2.48-8.13)	1.37 (0-2.84)	**0.004**, 0.346, **0.008**
Thrombopoietin	480.67 (334.25-608.13)	1013.39 (785.01-1253.54)	813.27 (696.13-922.02)	**<0.001**, **<0.001**, 0.285
TRAILR4	0 (0-2.45)	5.48 (3.45-15.64)	4.84 (2.06-8.25)	**0.004**, **0.036**, 0.249
TREM-1	7.61 (0-30.73)	47.33 (35.04-69.51)	14.3 (9.16-32.96)	**<0.001**, 0.254, **0.002**
bFGF	3250.94 (2080-4428.12)	687.85 (278.86-919.84)	317.59 (164.41-575.21)	**<0.001**, **<0.001**, 0.172
EGF	5.34 (3.67-7.21)	1 (0.33-2.57)	0.91 (0.54-1.25)	**0.003**, **<0.001**, 0.723

EGFR	1828.94 (1637.76-2741.6)	1152.51 (982.37-1564.14)	1248.74 (916.54-1462.7)	**<0.001**, **0.003**, 0.787
GDF-15	30.92 (25.98-43.16)	187.71 (107.44-426.93)	169.62 (111.88-302.34)	**<0.001**, **<0.001**, 0.755
HGF	405.89 (300.73-461.66)	177.31 (156.05-250.76)	153.8 (77.54-187.21)	**<0.001**, **<0.001**, 0.113
IGFBP-2	429.06 (298.35-657.38)	808.92 (403.5-1512.67)	1012.34 (718.34-1205.77)	**0.010**, **0.002**, 0.787
IGFBP-3	648.86 (349.57-1103.96)	2591.49 (1060.39-3952.26)	3295.19 (2087.64-4625.84)	**0.004**, **<0.001**, 0.368
Insulin	135.68 (102.86-159.6)	53.23 (0-104.07)	96.79 (52.91-150.74)	**0.003**, 0.180, 0.087
M-CSFR	118.56 (69.78-169.87)	319.59 (250.22-576.62)	239.84 (195.94-293.56)	**<0.001**, **<0.001**, 0.095
PDGF-AA	12.39 (9.1-17.36)	1.89 (0.66-3.46)	1.7 (1.41-2.29)	**<0.001**, **<0.001**, 0.851
VEGF-A	7.41 (6.42-10.89)	4.09 (3.19-5.69)	4.9 (4.21-5.89)	**0.002**, **0.009**, 0.573
CXCL13 (BLC)	0.08 (0-0.61)	2.72 (1.02-16.11)	2.03 (0.54-20.07)	**<0.001**, **0.017**, 0.632
CCL11 (Eotaxin-1)	0 (0-0.29)	3.66 (0.53-7.22)	9.79 (3.26-22.46)	**0.012**, **0.003**, 0.087
CCL24 (Eotaxin-2)	6.93 (5.2-14.26)	33.87 (14.05-84.89)	299.19 (91.9-582.69)	**0.001**, **<0.001**, **0.003**
G-CSF	0 (0-0)	78.72 (30.94-279.51)	36.08 (16.15-56.16)	**<0.001**, **<0.001**, 0.158
IL-1ra	0.14 (0-13.21)	29.03 (10.93-37.65)	34.72 (25.28-49.38)	**0.024**, **0.004**, 0.285
IL-4	0.14 (0-0.81)	0.3 (0.03-1.32)	2.34 (1.25-2.91)	0.524, **0.007**, **0.017**
IL-5	0.27 (0-1.1)	0 (0-1.56)	3.01 (0.69-5.17)	0.759, **0.017**, **0.013**
IL-10	0.39 (0.14-0.63)	0.39 (0-0.96)	1.78 (1.04-2.12)	1.000, **<0.001**, **<0.001**
IL-12p70	0.03 (0-0.14)	0.08 (0-0.23)	0.43 (0.28-0.66)	0.588, **0.003**, **0.002**
IL-13	0.11 (0-0.34)	0.29 (0-0.5)	0.88 (0.42-1.66)	0.286, **0.003**, **0.025**
IL-15	0 (0-5.01)	10.88 (0-42.62)	57.83 (29.13-82.73)	0.208, **0.001**, **0.008**
IL-17A	0.08 (0-0.39)	0.66 (0.32-1.53)	1.08 (0.68-1.23)	**0.006**, **<0.001**, 0.439
CCL2 (MCP-1)	12.48 (9.68-14.37)	75.8 (32.36-97.24)	42.26 (31.29-61.95)	**<0.001**, **<0.001**, 0.368
CCL15 (MIP-1δ)	2.17 (0.14-3.61)	11.59 (0.73-15.77)	10.28 (6.56-16.65)	0.057, **<0.001**, 0.632
PDGF-BB	23.97 (10.75-41.06)	8.04 (2.46-11.47)	6.37 (4.38-7.99)	**0.024**, **0.002**, 0.518
TNFRI	111.61 (50.02-131.63)	206.96 (160.96-308.87)	159.73 (85.65-198.15)	**<0.001**, 0.093, 0.113
TNFRII	287.01 (224.28-398.46)	818.35 (477.13-1449.62)	412.38 (203.91-686.44)	**0.003**, 0.418, 0.065

CD14	2062.31 (1748.39-2383.65)	4971.86 (4071.34-6423.26)	3938.16 (2716.8-4757.09)	**<0.001**, **0.009**, **0.039**
CEACAM-1	183.01 (159.99-201.84)	238.61 (190.6-342.18)	298.18 (247.29-592.78)	**0.031**, **<0.001**, 0.072
CD105 (Endoglin)	3048.71 (2840.73-3611.05)	2154.96 (1438.25-2856.68)	1514.3 (1264.28-2165.2)	**0.016**, **0.002**, 0.267
ErbB3	5891.85 (4846.56-7997.64)	1433.21 (541.37-1924.77)	1011.79 (121.97-2105.73)	**<0.001**, **<0.001**, 0.602
Flt-3 Ligand	13.24 (8.41-24.84)	3.91 (2.24-6.47)	2.48 (1.81-9.39)	**0.006**, **0.004**, 0.632
GITR (TNFRSF18)	2.99 (0-7.01)	16.06 (9-29.15)	7.72 (4.92-15.6)	**0.001**, 0.080, **0.035**
IL-17RA	9 (6.57-12.73)	14.98 (11.39-21.31)	20.7 (13.24-26)	**0.024**, **0.004**, 0.346
LIMPII	30.02 (16.56-35.36)	7.89 (2.82-12.82)	7.85 (1.93-11.34)	**0.001**, **<0.001**, 0.632
Lipocalin-2 (NGAL)	2673 (2263.17-2761.73)	3957.21 (3290.64-4334.54)	3261.41 (2693.88-3306.59)	**<0.001**, 0.107, **0.004**
L-Selectin	1267.05 (897.5-1701.16)	3263.37 (2183.15-3872.34)	2457.56 (1799.68-4108.86)	**<0.001**, **0.006**, 0.692
LYVE-1	149.16 (81.94-238.25)	450.46 (251.05-647.07)	313.42 (259.86-846.24)	**0.005**, **0.014**, 0.787
MICA	8.43 (5.58-12.35)	17.54 (13.41-24.65)	13.43 (12.23-17.41)	**0.004**, **0.025**, 0.172
RAGE	41.63 (24.44-74.78)	2.82 (1.35-10.37)	10 (5.33-16.28)	**<0.001**, **<0.001**, 0.095
TIM-1 (KIM-1)	1.03 (0-4.67)	11.91 (7.05-15.01)	6.15 (4.04-11.56)	**0.001**, **0.011**, 0.249
TRAILR3	0.79 (0.05-1.84)	24.59 (12.94-39.36)	45.71 (29.23-96.41)	**<0.001**, **<0.001**, 0.124
uPAR	15.97 (0-27.53)	320.44 (139.9-507.79)	210.83 (163.84-393.89)	**<0.001**, **<0.001**, 0.573

Data are expressed as medians (interquartile ranges).

* *P* value: NECRSwNP *vs.* Control, ECRSwNP *vs.* Control, and ECRSwNP *vs.* NECRSwNP, respectively. *P* values were obtained from the Mann-Whitney *U* test.

The significant values are indicated in bold.

Abbreviations: CXCL, C-X-C motif chemokine; CCL, C-C motif chemokine; GRO, growth-regulated alpha protein; MCP-2, monocyte chemotactic protein 2; MIP-3α, macrophage inflammatory protein 3 alpha; MSP, macrophage stimulating protein; PARC, pulmonary and activation-regulated chemokine; FcγRIIB/C, low affinity immunoglobulin gamma Fc region receptor II b/c; IL-2Rα, interleukin 2 receptor subunit alpha; IL-2Rβ, interleukin 2 receptor subunit beta; PDGF-AB, platelet-derived growth factor AB; SDF-1β, stromal cell-derived factor 1 beta; TGF-β2, transforming growth factor beta 2; TRAILR4, TNF-related apoptosis-inducing ligand receptor 4; TREM-1, triggering receptor expressed on myeloid cells 1; bFGF, basic fibroblast growth factor; EGF, epidermal growth factor; EGFR, epidermal growth factor receptor; GDF-15, growth differentiation factor 15; HGF, hepatocyte growth factor; IGFBP-2, insulin-like growth factor-binding protein 2; IGFBP-3, insulin-like growth factor-binding protein 3; M-CSFR, macrophage colony stimulating factor 1 receptor; PDGF-AA, platelet-derived growth factor AA; VEGF-A, vascular endothelial growth

factor A; BLC, B lymphocyte chemoattractant; G-CSF, granulocyte colony-stimulating factor; IL-1ra, interleukin 1 receptor antagonist; MCP-1, monocyte chemotactic protein 1; MIP-1δ, macrophage inflammatory protein 1 delta; PDGF-BB, platelet-derived growth factor BB; TNFRI, tumor necrosis factor receptor 1; TNFRII, tumor necrosis factor receptor 2; CEACAM-1, carcinoembryonic antigen-related cell adhesion molecule 1; ErbB3, receptor tyrosine-protein kinase erbB-3; Flt-3 Ligand, fms-related tyrosine kinase 3 ligand; GITR, glucocorticoid-induced tumor necrosis factor receptor related protein; TNFRSF18, tumor necrosis factor receptor superfamily member 18; IL-17RA, interleukin 17 receptor A; LIMPII, lysosomal integral membrane protein 2; NGAL, neutrophil gelatinase-associated lipocalin; LYVE-1, lymphatic vessel endothelial hyaluronic acid receptor 1; MICA, MHC class I polypeptide-related sequence A; RAGE, receptor for advanced glycation end products; TIM-1, T cell immunoglobulin mucin receptor 1 ; KIM-1, kidney injury molecule 1; TRAILR3, TNF-related apoptosis-inducing ligand receptor 3; uPAR, urokinase plasminogen activator surface receptor; ECRSwNP, eosinophilic chronic rhinosinusitis with nasal polyposis; NECRSwNP, noneosinophilic chronic rhinosinusitis with nasal polyposis.
